# Diagnostic value of acoustic radiation force impulse imaging for assessing superficial lymph nodes: A diagnostic accuracy study

**DOI:** 10.1097/MD.0000000000008125

**Published:** 2017-10-27

**Authors:** Fengjuan Zhang, Xinming Zhao, Xiaohui Ji, Ruoling Han, Ping Li, Min Du

**Affiliations:** a Department of Ultrasound, the Fourth Hospital of Hebei Medical University; b Department of Nuclear Medicine and Medical Imaging, the Fourth Hospital of Hebei Medical University, Hebei, Shijiazhuang, China.

**Keywords:** acoustic radiation force impulse, lymph node, shear wave velocity, sonography, virtual touch tissue imaging, virtual touch tissue quantification

## Abstract

The aim of this study was to assess the diagnostic value of acoustic radiation force impulse (ARFI) imaging for differentiating superficial lymph nodes.

Virtual touch tissue imaging (VTI) grade and shear wave velocity (SWV) were analyzed and compared in 97 patients (65 women, 32 men; mean age, 49 y; range, 23–72 y) with 97 lymph nodes [23 chronic nonspecific reactive lymph nodes (CLNs), 38 metastatic lymph nodes (MLNs), and 36 blood and lymphatic system diseases lymph nodes (BLLNs)]. The elastography characteristics in patients with CLNs, MLNs, and BLLNs were compared using the nonparametric Kruskal-Wallis test and Mann-Whitney *U* test for continuous variables and categorical variables. The diagnostic performance of VTI grade and SWV were evaluated using the area under the receiver operating characteristic curve (AUC).

The median of SWV of MLNs was significantly higher (2.90 m/s) than those of CLNs (2.15 m/s) and BLLNs (2.52 m/s). The VTI grade of MLNs was significantly higher than those of CLNs (*P* < .001) and BLLNs (*P* < .001). The sensitivity, specificity, accuracy, and AUC were 81.58%, 95.65%, 86.89%, and 0.904, respectively, at a cutoff level of grade IV for VTI grade in differentiating MLNs from CLNs, whereas those of SWV were 57.89%, 86.96%, 68.85%, and 0.752, respectively, at a cutoff level of 2.76 m/s.

The diagnostic performance of VTI grade was significantly higher than that of SWV in differentiating MLNs from CLNs. The diagnostic performance of VTI grade and SWV were lower intermediate in differentiating MLNs from BLLNs and in differentiating BLLNs from CLNs, and there was no significant difference between VTI grade and SWV. ARFI imaging may be a feasible method for differentiating MLNs from CLNs.

## 1 Introduction

Identifying the nature of the lymph nodes is the most important procedure in oncologic staging and classification because it directly affects the prognosis and treatment decision.^[1]^ Conventional sonography has become the preferred imaging method for screening for benign and malignant superficial lymph node.^[2]^ However, the conventional sonographic criteria for metastatic lymph nodes are still controversial.^[3–5]^ What's more, no single ultrasonography criterion for malignant lymph nodes had satisfactory sensitivity and specificity.^[6]^ A simple, reliable, noninvasive imaging method for categorizing lymph nodes is needed.

Ultrasound elastography is a promising modality for measuring the hardness of tissue. Conventional elastography for lymph nodes have utilized visual or software-assisted grading of color-coded strain elastograms or semiquantitative strain indices to estimate the tissue stiffness. Elasticity score and strain ratio are used for the interpretation of conventional elastography. It has played a certain role as a complementary tool in differentiating benign from malignant superficial lymph nodes,^[7–10]^ but there are some disadvantages with freehand compression and that these results were relative values that obtained by compared with a reference tissue such as muscle. The results of conventional elastography for lymph nodes are affected by the skills of the sonographer in vibrating or stressing the tissue.

Acoustic radiation force impulse (ARFI) imaging is a relatively new elastography technique that reduces operator dependency and improves reproducibility because of the addition of automated acoustic stress,^[11,12]^ instead of extra pressure exerted by sonographer. ARFI imaging includes virtual touch tissue imaging (VTI) and virtual touch tissue quantification (VTQ) that can respectively qualitatively and quantitatively evaluate the elasticity of the tissue. VTQ can quantitative measurement the absolute hardness of the tissue rather than relative values. The fundamental principle of ARFI imaging as described below. The ultrasound transducer launches short-duration and high-intensity acoustic pulses when VTI was activated. The acoustic pulses acted as extrinsic stress that pushed the tissue within the region of interest (ROI) to produce microdisplacement. The displacement is related to the elasticity of the tissue, the greater the elasticity of the tissue within ROI, the greater the displacement.^[13]^ VTI image is displayed as a grey-scale image, the softer the tissue is, the larger the displacement is, the brighter the image is, on the contrary, the stiffer the tissue is, the smaller the displacement is, the darker the image is. Tissue within the sampling box generates transverse shear wave when it is pushed by the high-intensity and short-duration acoustic pulses, which is produced by ultrasound transducer. The shear wave velocity (SWV) is tracked and calculated by using correlation-based methods. The SWV is expressed in “meters/second” (m/s), and the SWV is related to the elasticity of the tissue, the stiffer the tissue is, the faster the shear wave propagates, on the contrary, the softer the tissue is, the slower the shear wave propagates. VTQ is performed by measuring the SWV of the tissue within the sampling box. VTQ can get specific values of SWV that are associated with elasticity; therefore, VTQ is a quantitative measurement method of tissue elasticity.

To our knowledge, a few studies regarding the diagnostic performance of VTI classification, area ratio of VTI images of lymph nodes to 2-dimensional ultrasound images and SWV for lymph nodes have been conducted; however, whether VTI grade for malignant blood and lymphatic system diseases have difference with those of metastatic lymph nodes and chronic nonspecific reactive lymph nodes is uncertain. The purpose of this study was to assess and compare the diagnostic value of the SWV and VTI grade of superficial metastatic lymph nodes, chronic nonspecific reactive lymph nodes and malignant blood and lymphatic system diseases lymph nodes, with histopathologic analysis of surgical and core needle biopsy specimens as the reference standard. The accuracy of conventional sonographic criteria for malignant lymph nodes has been widely reported in previous literatures; therefore, this part content was not included in this study.

## 2 Materials and methods

### 2.1 Patients

Informed consent was obtained from all patients, and the study was performed in accordance with the ethics guidelines of the Helsinki Declaration and approved by the local ethics committee of Hebei Medical University. From October 2015 to March 2016, 108 consecutive patients with suspicious superficial lymph nodes who were detected by conventional sonography were recruited for ARFI imaging and they were assigned a number according to the sequence of registration. The enrollment criteria of patients were as follows: the cortical thickness of lymph nodes were at least 0.5 cm; the minimum diameter of lymph nodes was greater than or equal to 0.6 cm to ensure that the peripheral tissue was not included in the sample frame of VTQ, which with the sample volume of 0.5 × 0.6 cm and the maximum diameter was less than or equal to 2 cm to ensure that there was enough peripheral tissue in VTI whose maximal region of interest of 2 × 3 cm; neither clinical treatment nor fine needle aspiration (FNA) biopsy nor core needle biopsy of the nodules was performed before ultrasound examination. The latter criterion was included because these invasive procedures would have changed the original hardness of lymph nodes. The effective range for the SWV is 0 to 9 m/s. Higher SWV indicated stiffer tissue. Value beyond these range was displayed as “x.xx m/s” that was deemed invalid measurements. Eleven patients were excluded from the study because 10 SWV measurements of each target lymph node were all expressed as “x.xx m/s.” Finally, 97 patients (65 women and 32 men; mean age, 49 y; range, 23–72 y) with 97 superficial lymph nodes were evaluated in the study. All target lymph nodes were located on body surface after conventional sonography and the distance from the target node to the skin was recorded to correlate with pathology results when there were multiple nodes within one patient. Ninety-seven lymph nodes underwent ultrasound-guided core needle biopsy instead of fine-needle aspiration biopsy because most patients need immunohistochemical to determine pathological type, and 42 of them were surgically removed, and all nodes got clear histopathological results.

### 2.2 Conventional sonography and ARFI imaging

Both conventional sonography and ARFI imaging were performed with the same S2000 ultrasound system (Siemens Medical Solutions, Mountain View, CA) with a 6 to 15 MHz linear transducer for conventional sonography and a 4 to 9 MHz linear transducer for ARFI imaging. Each patient was placed in the supine position. All of the lymph nodes were observed with conventional sonography first. We considered lymph nodes abnormal when conventional sonography findings revealed at least one of the following criteria: cortical echo decreased, increased, or heterogeneous, cortex thicken (>0.3 cm) or loss of hilar fat, calcification or cystic degeneration, long- axis to short-axis diameter ratio < 2.0. If there was only 1 abnormal lymph node, we used it for ARFI imaging. When patients had multiple abnormal lymph nodes, we chose the node with the most abnormal features on conventional sonography as representative, which was isolated with more surrounding tissue as controls, close to the body surface, away from the arteries, bone and big muscles. The following features of target lymph nodes were observed: size, echogenicity, margin, shape, and long- axis to short-axis diameter ratio. After that, the radiologist located the probe vertical to the skin. To obtain appropriate images, the probe was applied with minimal pressure to make complete contact with the lymph node. The longitudinal plane of the target lymph node was displayed on conventional sonography first, then a rectangular ROI that was used for VTI acquisition presented on the screen when VTI mode was activated, then the ROI was adjusted to the maximum range to include the node and adjacent tissue. Some cervical lymph nodes may generate obvious displacement when affected by arterial fluctuation and breathing; therefore, some patients were asked to avoid swallowing and hold their breath during ARFI imaging. The update button was pressed when patients were ready, then the VTI image was produced on the right of the corresponding B-mode scan. This procedure was repeated 3 times, and one image with best defined borders was chosen to save as research data. For the VTI image, bright regions indicated tissue were more softer than dark regions. The grayscale value in VTI imaging was classified into black or white through the comparison between the lymph node and the surrounding tissue. Depending on the proportion of black and white shown in the lymph node, referring to Xu's VTI grading for thyroid nodules,^[14]^ VTI images of lymph nodes were divided into 6 grades. A higher grade indicated stiffer tissue.

The patients’ position and the probe's location remained unchanged. A sampling box with fixed dimension of 0.5 × 0.6 cm presented on the screen when VTQ function was chosen. The sampling box was placed in the hypoechoic regions or suspicious regions of the lymph node. The VTQ function was initiated when patients were ready according to the radiologist's requirements, then SWV was displayed on the screen, which was expressed in meters per second (m/s). Ten valid measurements were performed in different hypoechoic regions or suspicious regions of the lymph node, and the calcified or cystic portions were avoided as far as possible. The mean value of ten valid measurements was used for the analysis.

VTI and VTQ were performed sequentially for odd-numbered patients, whereas VTQ and VTI were performed sequentially for even-numbered patients. Body surface marking was marked for correlation with pathology results when there were multiple lymph nodes within the same position. The conventional sonography, ARFI imaging, and body surface marking were performed by one radiologist (F.J.Z.) with 7 years of experience in lymph nodes sonography and 1 years of experience in lymph nodes ARFI imaging.

The mean SWV of each lymph node was calculated and the VTI image was graded in the same setting and in a blind manner independently by 2 radiologists (R.L.H. and X.H.J.) with more than 10 years of experience in lymph nodes sonography and 2 years of experience in lymph nodes ARFI imaging. In cases of discord in the evaluation of the VTI grade between the 2 radiologists, a third radiologist (X.M.Z, with 15 years of experience in lymph nodes sonography, 3 years of experience in lymph nodes ARFI imaging) reviewed the images to make the final decision. In addition, the third radiologist confirmed the mean SWV of each lymph node. The identities of the patients and the clinical and pathology results were not available to these 3 radiologists.

### 2.3 Pathology diagnoses

According to the clinical requirements or the intentions of the patients, all target lymph nodes in our study were confirmed histologically, and all pathological diagnoses were made by a pathologist experienced in lymphatic system disorders pathological examination.

### 2.4 Statistical analysis

The elastography characteristics in patients with chronic nonspecific reactive lymph nodes (CLNs), malignant blood and lymphatic system diseases lymph nodes (BLLNs), and metastatic lymph nodes (MLNs) were compared using the nonparametric Kruskal-Wallis test and Mann-Whitney *U* test for continuous variables and categorical variables. The level of significance was set at 0.05 for all tests. The above analyses were performed using SPSS software (version 13.0; SPSS, Chicago, IL). The diagnostic performance of VTI grade and SWV were evaluated using the area under the receiver operating characteristic curve (AUC). The diagnostic performance was regarded as low (AUC = 0.5–0.7), moderate (AUC = 0.7–0.9), or high (AUC > 0.9). The comparison of AUCs between VTI grade and SWV in differentiating MLNs from CLNs was performed using MedCalc statistical software (version 13.0.0), and in differentiating MLNs from BLLNs and in differentiating BLLNs from CLNs.

## 3 Results

### 3.1 Patients

From October 2015 to March 2016, 108 patients were recruited. Eleven patients were excluded from the study because 10 SWV measurements of each target lymph node were all expressed as “x.xx m/s.” Finally, 97 patients (65 women, 32 men; mean age, 49 y; range, 23–72 y) with 97 lymph nodes were evaluated in the study.

### 3.2 Histopathological findings

The histopathological results revealed 23 chronic nonspecific reactive lymph nodes (CLNs), 36 malignant blood and lymphatic system diseases lymph nodes (BLLNs, 2 chronic granulocytic leukemia, 1 chronic lymphocytic leukemia, 15 Hodgkin's lymphoma, 18 non-Hodgkin's lymphoma) and 38 metastatic lymph nodes (MLNs). MLNs comprised as follows: 3 adenocarcinoma of the lung, 3 squamous cell carcinoma of the lung, 3 small cell carcinoma of the lung, 5 squamous cell carcinoma of the esophagus, 1 kidney cancer, 1 small bowel cancer, 3 papillary thyroid cancer, and 19 breast carcinoma.

### 3.3 SWV

**Figure 1:**
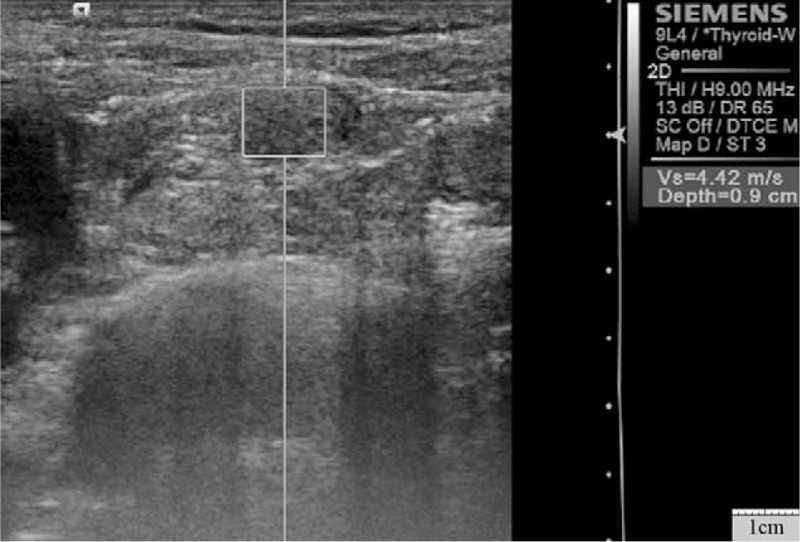
Virtual touch tissue quantification image of metastatic lymph node in a 47-year-old woman with small cell lung cancer. The shear wave velocity was 4.42 m/s.

**Figure 2:**
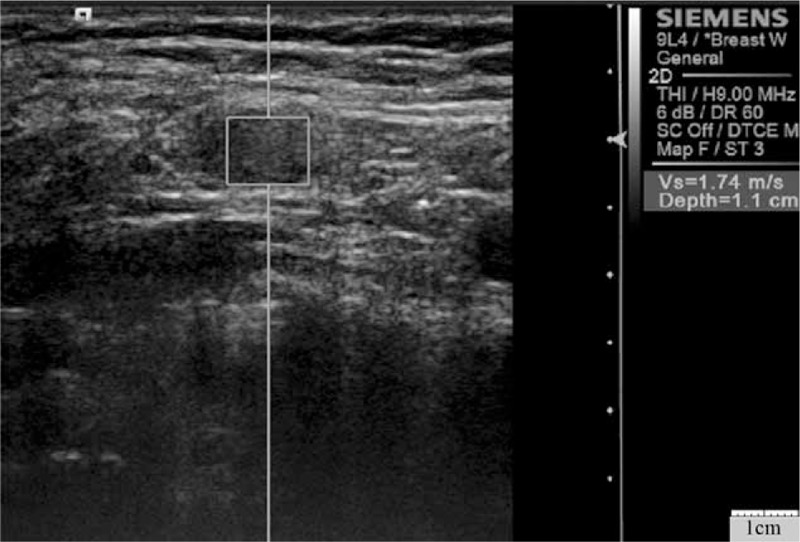
Virtual touch tissue quantification image of chronic nonspecific reactive lymph node in a 46-year-old woman. The shear wave velocity was 1.74 m/s.

**Figure 3:**
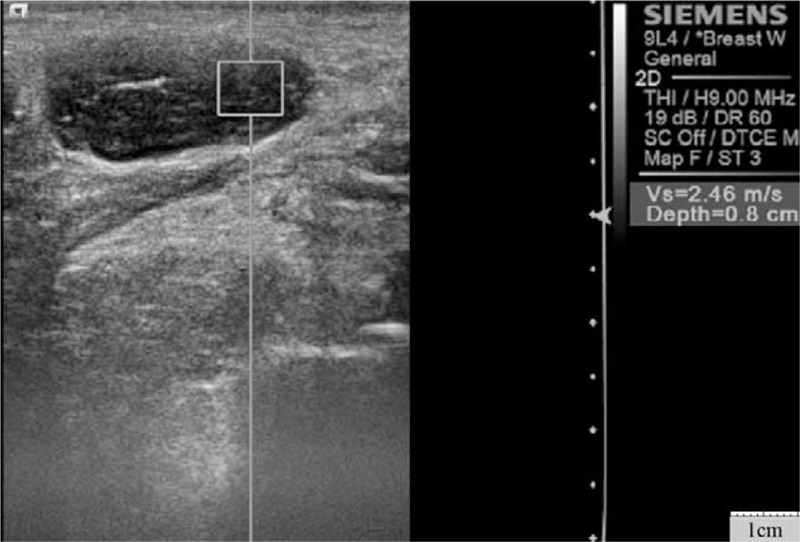
Virtual touch tissue quantification image of chronic lymphocytic leukemia in a 58-year-old man. The shear wave velocity was 2.46 m/s.

The median of SWV of CLNs, MLNs, and BLLN was 2.15 m/s, 2.90 m/s, and 2.52 m/s, respectively. The SWV of MLNs (Fig. 1) was significantly higher than that of CLNs (Fig. 2, *P* < .05) and that of BLLNs (Fig. 3, *P* < .05). The cutoff level for SWV for differentiating MLNs from CLNs was estimated to be 2.76 m/s. Using the ROC curves with this cutoff value, the SWV distinguished MLNs from CLNs with a sensitivity of 57.89%, specificity of 86.96%, and accuracy of 68.85%. The best cutoff value of SWV for differentiating MLNs from BLLNs was 3.44 m/s, giving sensitivity of 39.47%, specificity of 94.44%, and accuracy of 66.22%. The median of BLLNs has no significant difference with that of CLN. SWV differentiating BLLNs from CLNs with sensitivity of 63.89%, specificity of 69.57%, and accuracy of 66.10% at a cutoff level of 2.35 m/s.

### 3.4 VTI grade

**Figure 4:**
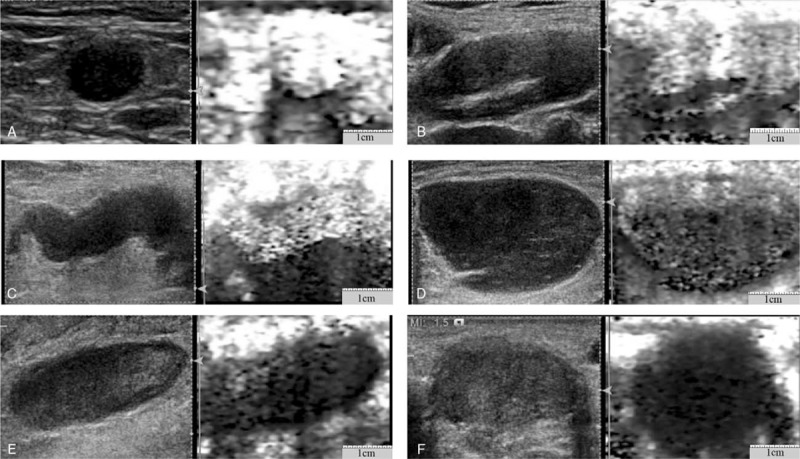
Virtual touch tissue imaging grades of lymph nodes (I–VI). A, Grade I: the lymph nodes are displayed homogeneously in white or with a little point-like black. B, Grade II: almost the whole lymph nodes is displayed in white, with a small amount of black. C, Grade III: the black and white portions in the whole lymph nodes are almost the same. D, Grade IV: almost the whole lymph nodes is displayed in black, with a small amount of white. E, Grade V: the lymph nodes is displayed almost in black, with a little point-like white. F, Grade VI: the whole lymph nodes is displayed homogeneously in black.

VTI images of lymph nodes were divided into six grades (Fig. 4, Table 1). The VTI grade was significantly higher in MLNs (MLNs vs CLNs, *P* < .05, MLNs vs BLLNs, *P* < .05). When displayed as VTI images, 82.61% of the CLNs and 72.22% of the BLLNs showed VTI grades less than or equal to grade III, whereas only 18.42% of the MLNs showed VTI grades less than or equal to grade III; 81.58% of the MLNs showed VTI grades greater than or equal to grade IV, whereas only 17.39% of the CLNs and 27.78% of the BLLNs showed VTI grades greater than or equal to grade IV (Table 1). VTI grade using grade IV had 81.58% sensitivity, 95.65% specificity, and 86.89% accuracy for differentiating MLNs from CLNs. The best cutoff value of VTI grade for differentiating MLNs from BLLNs was grade IV, giving sensitivity of 81.58%, specificity of 72.22%, and accuracy of 77.03%. The VTI grade of BLLNs had no significant difference with CLNs (*P* > .05). The VTI grade for differentiating BLLNs from CLNs with sensitivity of 27.80%, specificity of 95.70%, and accuracy of 54.24% at a cutoff level of grade IV.

**Table 1:**
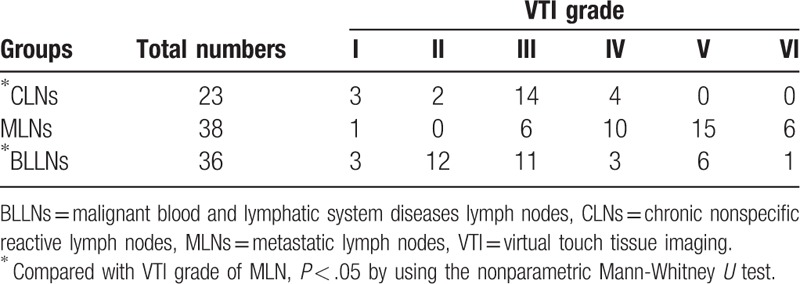
VTI grades of different lymph node diseases.

### 3.5 The diagnostic performance of VTI grade and SWV

**Figure 5:**
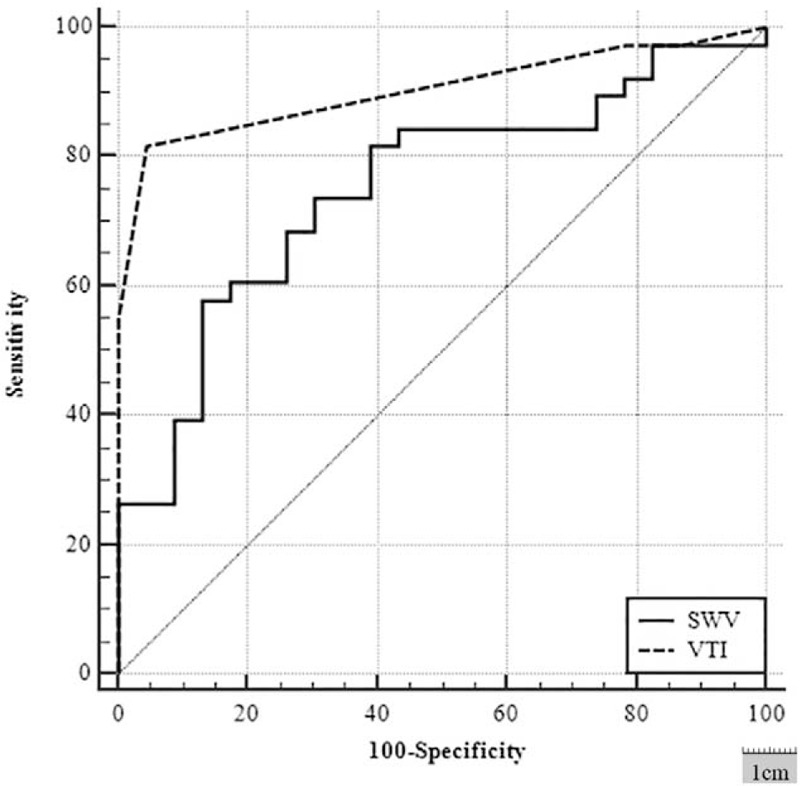
Receiver operating characteristic curves of virtual touch tissue imaging grade and shear wave velocity for differentiating metastatic lymph nodes from chronic nonspecific reactive lymph nodes. The area under the curve was 0.904 and 0.752, respectively.

The AUCs of VTI grade were 0.904 (95% CI, 0.802−0.965), 0.799 (95% CI, 0.689–0.883), and 0.519 (95% CI 0.385−0.651), respectively, for differentiating MLNs from CLNs, differentiating MLNs from BLLNs, and differentiating BLLNs from CLNs, whereas the corresponding AUCs of SWV were 0.752 (95% CI 0.624−0.853), 0.667 (95% CI, 0.548–0.772), and 0.638 (95% CI, 0.502−0.759), respectively. The diagnostic performance of VTI grade was higher than that of SWV in differentiating MLNs from CLNs (Fig. 5, *P* < .05), whereas no significant different in differentiating MLNs from BLLNs (*P* *>* .05), and in differentiating BLLNs from CLNs (*P* > .05, Table 2).

**Table 2:**
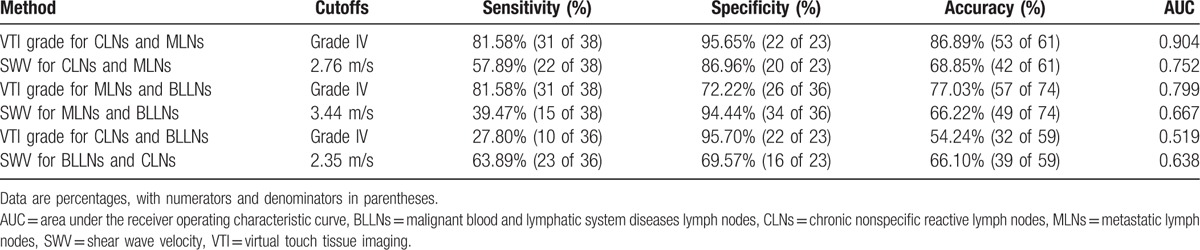
Diagnostic value of VTI grade and SWV.

## 4 Discussion

ARFI imaging is a relatively new imaging method that can be used to noninvasively assess the stiffness of target tissues. ARFI imaging has achieved excellent diagnostic performance in differentiating benign malignant thyroid nodules and breast lesions. In previous studies, Fujiwara et al^[11]^ have reported that a SWV cutoff value of 1.9 m/s enabled distinction between metastatic lymph nodes and benign lymph nodes with 95.0% sensitivity, 81.8% specificity, 88% accuracy, and 0.923 AUC. Che et al^[15]^ classified VTI images of lymph nodes into 2 VTI patterns by comparing with the surrounding tissue or muscles: slightly darker or the same brightness and obviously darker. It predicted malignant lymph nodes with the sensitivity of 93.3%, specificity of 91.7%, and accuracy of 92.5%. These studies suggested that VTI and SWV could be two valuable tools for assessment of benign and malignant lymph nodes.

In this study, the criterion of VTI grade IV had 81.58% sensitivity, 95.65% specificity, and 0.904 AUC in differentiating MLNs from CLNs, whereas the criterion of SWV > 2.76 m/s only had 57.89% sensitivity, 86.96% specificity, and 0.752 AUC. Compared with the above 2 reports, our study results disclosed the VTI grade and SWV have the lower sensitivity in differentiating MLNs from CLNs. The discrepancy may be explained by the different patients with different histologic types or a small study sample size. The AUC showed that the diagnostic power of VTI grade was high, which indicated that VTI grade may add potentially valuable information that may improve the differential diagnosis between MLNs and CLNs. In addition, the results also indicated that VTI grade may be more useful than SWV in differentiating MLNs from CLNs. In the study, no one CLNs showed grade V and grade VI, which suggested that VTI grade V and grade VI may be useful in excluding CLNs. When VTI images showed grade V and grade VI, further examination should be performed actively instead of watchful waiting policy to avoid misdiagnosis. The sensitivity of SWV is relatively low perhaps because of the following reasons: the SWV values showed as “x.xx m/s” were eliminated as invalid value; however, it may represent the lymph node is too hard to measure the SWV, therefore, the mean SWV of MLNs may be decreased; it may be related to the pathologic type of MLNs, adenocarcinoma is deemed to be soft,^[15]^ it may be related to the number of cancer cells and the proportion of metastasis, for example local invasion.

In the study, ARFI imaging revealed higher VTI grade and SWV for MLNs than for CLNs and for BLLNs, and whether VTI grade or SWV suggested that the stiffness of BLLNs was similar to the CLNs. The cortex of lymph node is damaged first when the cancer cells metastasize to the lymph node, then the cancer cells and the interstitial cells proliferate, the cancer cells will break through the lymph node capsule and infiltrate surrounding tissue in late period, therefore, the majority of MLNs are harder. The main pathological changes of CLNs are lymphocyte proliferation lymphoid follicles hyperplasia and germinal center expansion. Normal lymph node structure disappears and a large number of abnormal lymphocytes appear are the main pathological changes of BLLNs. Both CLNs and BLLNs have no obvious fibrosis and consist mostly of cell tissue, so the stiffness may be relatively soft.

A cutoff value of 7.302 m/s with 88.5% sensitivity and 81.5% specificity for differentiation of metastatic lymph nodes from lymphoma using SWV was reported.^[16]^ However, in this study, the sensitivity and specificity was 39.47% and 94.44% respectively at the cutoff value of 3.44 m/s.

The AUC of VTI grade and SWV for differentiating BLLNs from CLNs was 0.519 and 0.638, respectively. For ARFI imaging, the differential diagnosis between BLLNs and CLNs is a diagnostic challenge. Hence, conventional sonography was especially important, and it should be used as an underlying diagnostic pattern.

The diagnostic performance of VTI grade and SWV in differentiating BLLNs from MLNs is medium level. The BLLNs and the MLNs all belonged to malignant tumor, but the elastic characteristics were different. In general, the stiffness of MLNs was higher than that of BLLNs, ARFI may be helpful in the selection of suspicious lymph nodes that should be examined at ultrasound-guided percutaneous biopsy or surgical removal for accurate preoperative staging and individual therapy selection for patients with abnormal lymph nodes.

The same lymph node has different stiffness during different develop stage; therefore, there are certain overlaps in the stiffness among different kinds of lymph nodes. The VTI images of BLLNs were capricious, and they covered entire VTI grade; likewise, the variation range of SWV are extensive, therefore, there was no apparent tendency can be summarized, and these may lead to the above results. However, on the basis of conventional ultrasound diagnostic, combine the clinical data of patients and the results of ARFI, the diagnostic accuracy of lymph nodes can be further improved.

The instrument displayed the SWV as “x.xx m/s” when the value was out of the measurement range. Gallotti et al^[17]^ found the SWV to be displayed as “x.xx m/s” when the tissue inside the region of interest was heterogeneous or had a liquid component.^[17]^ This may be because heterogeneous tissues absorb most of the ultrasound energy and make shear wave measurement impracticable, and liquid can generate only an undetectable shear wave with the system. Eleven lymph nodes were excluded in the study because 10 measurement results were all shown as “x.xx m/s.” Ten cases were confirmed to be MLNs and the other was confirmed to be BLLNs after surgery, thus, “x.xx m/s” might serve as an indicator of malignant lymph nodes.

Technical limitations for ARFI imaging are described. First, the pulsation of the peripheral artery might affect the ARFI measurement when the lymph node is close to the peripheral artery. Second, the measurement range of SWV for this machine was 0 to 9 m/s, values outside this range were displayed as “x.xx m/s,” thus, the true stiffness of the lymph node was uncertain. Third, the fixed size and shape of sample frame of VTQ affected the accuracy of sampling. Future improvement of this application is needed.

Study limitations are mentioned here. First, this pilot study was made at a single institution, our sample series was relatively small, so it was not possible to perform a subgroup analysis of histologic type, although it may influence ARFI result. The diagnostic value of ARFI imaging in patients with different histologic types lymph node should be determined in future large scale sample studies. Second, the study cohort is not a representation of the general population because it only represents those who were scheduled to resect primary tumor or perform coarse needle biopsy for lymph node in the tumor hospital, so selection bias may be present in the study. Our institution is not only a tertiary tumor hospital, but also a diagnosis and treatment center of lymphoma; therefore, the malignancy rate is inevitably high. Finally, we did not compare conventional sonography with the ARFI imaging. Although the results of the current study exhibited the significant value of VTI grade in differentiating MLNs from CLNs, ARFI imaging provide additional diagnostic value to conventional ultrasound findings remains unknown. However, we believe that our findings provide important background data for future evaluations of the added value of ARFI imaging in larger populations.

## 5 Conclusions

VTI grade of ARFI imaging is a feasible modality for differential diagnosis between MLNs and CLNs and the diagnostic power is higher than that of SWV. Although the mean SWV of MLNs was found to be significantly higher than that of CLNs, there is still a significant false-negative rate, the invalid SWV measurements were eliminated that significantly influence the true SWV value of MLNs. ARFI imaging for distinguishing BLLNs from MLNs or CLNs are still a challenge. The usefulness and added value of ARFI imaging for evaluating superficial lymph nodes should be further evaluated in large multicenter studies.
